# Multi-Object Tracking in Heterogeneous environments (MOTHe) for animal video recordings

**DOI:** 10.7717/peerj.15573

**Published:** 2023-06-26

**Authors:** Akanksha Rathore, Ananth Sharma, Shaan Shah, Nitika Sharma, Colin Torney, Vishwesha Guttal

**Affiliations:** 1 Centre for Ecological Sciences, Indian Institute of Science, Bangalore, India; 2 Department of Electrical Engineering, Indian Institute of Technology, Bombay, Mumbai, India; 3 Department of Ecology and Evolutionary Biology, University of California, Los Angeles, Los Angeles, United States of America; 4 School of Mathematics and Statistics, University of Glasgow, Glasgow, United Kingdom

**Keywords:** Animal behaviour, Automated tracking, Computer vision, Convolutional neural network, Machine learning, Multi-animal tracking, Tracking in natural habitat

## Abstract

Aerial imagery and video recordings of animals are used for many areas of research such as animal behaviour, behavioural neuroscience and field biology. Many automated methods are being developed to extract data from such high-resolution videos. Most of the available tools are developed for videos taken under idealised laboratory conditions. Therefore, the task of animal detection and tracking for videos taken in natural settings remains challenging due to heterogeneous environments. Methods that are useful for field conditions are often difficult to implement and thus remain inaccessible to empirical researchers. To address this gap, we present an open-source package called Multi-Object Tracking in Heterogeneous environments (MOTHe), a Python-based application that uses a basic convolutional neural network for object detection. MOTHe offers a graphical interface to automate the various steps related to animal tracking such as training data generation, animal detection in complex backgrounds and visually tracking animals in the videos. Users can also generate training data and train a new model which can be used for object detection tasks for a completely new dataset. MOTHe doesn’t require any sophisticated infrastructure and can be run on basic desktop computing units. We demonstrate MOTHe on six video clips in varying background conditions. These videos are from two species in their natural habitat—wasp colonies on their nests (up to 12 individuals per colony) and antelope herds in four different habitats (up to 156 individuals in a herd). Using MOTHe, we are able to detect and track individuals in all these videos. MOTHe is available as an open-source GitHub repository with a detailed user guide and demonstrations at: https://github.com/tee-lab/MOTHe-GUI.

## Introduction

Video-recording of animals is becoming a norm in behavioural studies of space-use patterns, behavioural ecology, neuroscience, and field biology (Gonzalez et al., 2016; Mersch, Crespi & Keller, 2013; Tuci et al., 2019; Katz et al., 2011; Jhawar et al., 2020; Tuia et al., 2022). High-resolution images from aerial photographs and videos can also be used for animal census (Torney et al., 2019; Hodgson et al., 2018; Chabot, Craik & Bird, 2015; Tuia et al., 2022). We often need to extract behavioural or ecological information from these videos in order to analyse the data; for example, count of animals, areas covered by vegetation, the spatial position of individuals, postures or behavioural states of the individuals, *etc*. (Lauer et al., 2022; Pereira et al., 2022). For some of the observations, such as behavioural states and events, watching the videos might be sufficient. However, extracting spatial information such as coordinates and movement trajectories of a large number of animals can be time-consuming, tedious and often not feasible. The efficiency of performing such tasks manually often increases dramatically with increasing dataset size. Therefore, increasingly, automated tools are being developed to detect and track animals (Pérez-Escudero et al., 2014; Risse et al., 2017a; Mönck et al., 2018; Sridhar, Roche & Gingins, 2018; Rodriguez et al., 2018; Yamanaka & Takeuchi, 2018; Itskovits et al., 2017; Walter & Couzin, 2021; Nakagawa et al., 2022).

Most of the tools developed so far work best in controlled conditions. For example, Panadeiro et al. (2021) reviewed 28 openly source packages for animal tracking. They concluded that only five of those packages are suitable for detecting and tracking multiple unmarked animals  (Xu & Cheng, 2017; Rice et al., 2020; Rodriguez et al., 2018; Pérez-Escudero et al., 2014; Romero-Ferrero et al., 2019); the user documentations of these methods further reveals that these methods were tested and demonstrated only for videos taken in homogeneous backgrounds in laboratory conditions. Tracking animals from videos recorded in natural settings poses many challenges  (Kellenberger, Tuia & Morris, 2020; Tuia et al., 2022; Koger et al., 2023). These challenges include: variability in lighting conditions, camera vibration, disappearance and appearance of animals across video frames, and heterogeneous backgrounds. Under such conditions, existing tools which rely on traditional computer vision techniques such as image subtraction, colour thresholding, feature mapping, *etc.*, do not perform well. Therefore, many object detection tools in ecology that use these computer vision algorithms, although efficient for videos taken under controlled conditions, are likely to fail to detect or track animals in natural settings (Dell et al., 2014; Sridhar, Roche & Gingins, 2018; Bewley et al., 2016; Tuia et al., 2022; Koger et al., 2023).

One technique known to be efficient in solving detection problems in heterogeneous backgrounds is the use of convolutional neural networks (CNN) (Szegedy, Toshev & Erhan, 2013; Bowley et al., 2016; Norouzzadeh et al., 2018; Girshick, 2015; Ren et al., 2015). Despite the promise offered by CNN-based algorithms for object detection in heterogeneous environments, only a few adaptations of them are available in the context of animal tracking (Rastegari et al., 2016; Rey et al., 2017; Kellenberger, Marcos & Tuia, 2018; Risse et al., 2017b; Torney et al., 2019; Graving et al., 2019; Koger et al., 2023; Redmon et al., 2016; Redmon & Farhadi, 2018; Ren et al., 2015; Xu & Cheng, 2017; Ferreira et al., 2020). The few available algorithms for object detection in heterogeneous environments usually require high-performance computing units or cloud computing. Further, implementation often requires reasonable proficiency in computer programming together with a great amount of customization. Hence, there is a need for a relatively-easily customizable end-to-end application that automates the task of object detection and is usable even on simple desktop machines. Lack of an integrated end-to-end pipeline that allows users to perform data annotation to detection and tracking could be a major hindrance for adopting the latest advances in visual tracking for analysing empirical datasets, especially in the context of videos taken in the natural conditions in the field. To address this lacuna, we propose an end-to-end pipeline that uses a deep-learning approach.

Here, we provide an open-source package, Multi-Object Tracking in Heterogeneous environment (MOTHe), that can run as a graphical user interface (GUI) app within a Python environment. The functionalities include the generation of the training dataset, multi-object detection, and track linking across frames. The package can be customised for different datasets and can run on relatively basic desktop units. For a new dataset, users can generate training data using a semi-automated ‘drag and click’ functionality; a new model can be trained using these data. MOTHe can detect multiple individuals in heterogeneous backgrounds *i.e.,* videos recorded in a species’ natural habitat. It uses a colour thresholding approach followed by a CNN architecture to detect and classify objects within images, allowing a relatively fast training of the network even on generic desktop computing units. We demonstrate the application on six video clips from two species (wasps on the nests and antelope herds in four different types of habitats). These videos were recorded in natural and semi-natural settings having background heterogeneity and varying lighting conditions. We provide an open-to-use Github repository (https://github.com/tee-lab/MOTHe-GUI) along with a detailed user guide for the implementation.

## Materials & Methods

In this section, we present a broad overview of the features and principles on which MOTHe works. MOTHe is a python-based library and it uses a convolutional neural network (CNN) architecture for object detection. CNNs are specific types of neural network algorithms designed for tasks such as classification or object detection within images. Our CNN consists of three convolutional and two dense layers. The number of nodes for the convolutional layers is 64 whereas it is 96 to 128 for the dense layers. On the top of each convolutional layer, we use an activation function and a pooling layer; we refer the reader to Supplementary Material Section 1.2 for further details.

### Working principle & features

For the classification task, the CNN takes a digital image as an input and processes pixel values through a network and assigns a category to the image. To achieve this, CNN is trained *via* a large amount of user-labelled training data and learning algorithms; this procedure enables the network to learn features of objects of interest from the pool of training data. Once the CNN models are trained, these models can be used to identify objects in new datasets (Dhruv & Naskar, 2020). In the context of tracking multiple animals in a video, an object detection task involves identifying locations and categories of objects present in an image. MOTHe works for 2-category classification of objects, *e.g.*, animal and background.

**Figure 1:**
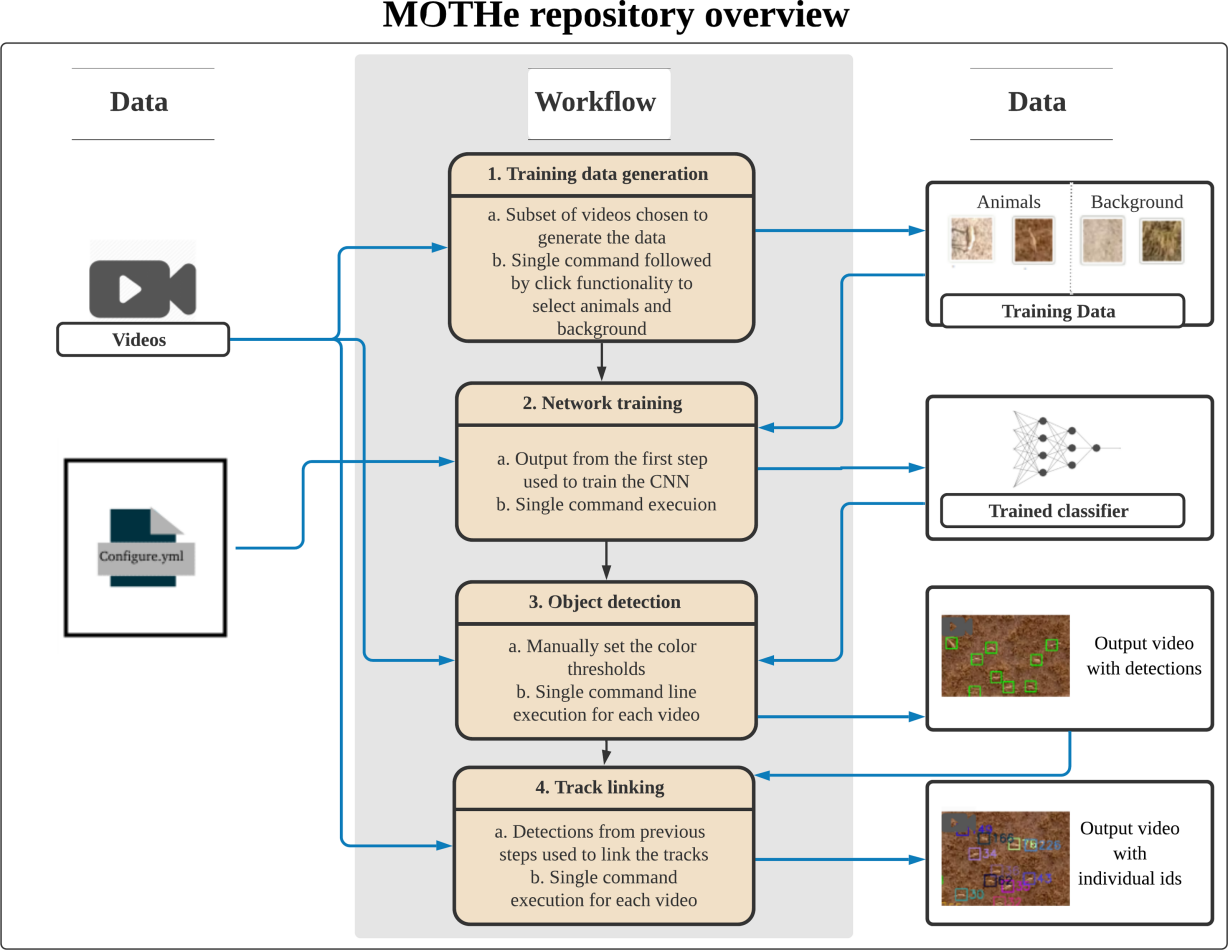
The layout of our GitHub repository. A configuration file is generated in the first step, which maintains directory paths and parameter values used by subsequent modules. Tracking happens in two steps—first, we need to train the network on training dataset; second, object detection is done using the trained CNN on the image. Each step here is a separate module that can be run by users. Black arrows represent the directional flow of executable files. Blue arrows represent input/output flow of data in the modules.

MOTHe is divided into four independent modules (see Fig. 1):

(i) **Generation of training dataset**—Dataset generation is a crucial step in object detection and tracking. In this step, we provide a graphical interface for data generation. Users select the “generate data” function in the GUI application to extract images for the two categories *i.e.,* animal and background. It allows users to crop regions of interest by simple clicks over a graphical user interface and saves the images in appropriate folders. On each run, users can input the category for which the data will be generated and specify the video from which images will be cropped. Outputs from this module are saved in two separate folders: one containing images of animals (yes) and the other containing background (no).

(ii) **Network training**—The network training module is used to create the network and train it using the dataset generated in the previous step. Users select the “train” function in the GUI application to perform the training. Once the training is complete, the training accuracy is displayed and the trained model (classifier) is saved in the repository. The accuracy of the classifier is dependent on how well the network is trained, which in turn depends on the quality and quantity of training data (see section “How much training data do I need?” on the repository help page). Various tuning parameters of the network, *e.g.,* the number of nodes, size of nodes, convolutional layers, *etc.* are fixed to render the process easy for the user.

(iii) **Object detection**—To perform the detection task, we first need to identify the areas in an image where the object can be found, this is called localisation or region proposal. Then we classify these regions into different categories (e.g., whether an animal or background?), this step is called classification. The localisation step is performed using an efficient thresholding approach that restricts the number of individual classifications that need to be performed on the image. The classification at each location is then performed using the trained CNN generated in the previous module. The outputs, detected animals, are in the form of CSV files that contains locations of identified animals in each frame.

(iv) **Track linking**—This module assigns unique IDs to the detected individuals and generates their trajectories. We use a standard approach for track linking that uses a Kalman filter to predict the next location of the object and the Hungarian algorithm to match objects across frames (Sahbani & Adiprawita, 2016; Hamuda et al., 2018). This script can be run once the detection output is generated in the previous step. The output is a CSV file that contains individual IDs and locations in each frame. Video output with unique IDs on each individual is also generated.

To make MOTHe fast to train and run on new videos we use grayscale-thresholding as the localisation or region proposal step (Taghizadeh & Chalechale, 2022). As discussed earlier, colour thresholding or grayscale thresholding has limitations in case of complex backgrounds, low object-background contrast and confusing objects, posing a trade-off between missing animals or falsely detected background objects. To utilise thresholding as a localisation step, we err on the side of false detections *i.e.,* detect a higher number of keypoints potentially containing animals as well as other background objects. We then use these keypoints as the regions of interest and run the classification over the images generated from the keypoints. This step reduces the computation time compared to a sliding window approach (Gould, Gao & Koller, 2009). Furthermore, overfitting is an important issue in machine learning. The use of a compact CNN architecture has the advantage of requiring smaller training datasets and is less prone to overfitting than deeper networks (see section 1.2 in the Supplementary Material for the details of network architecture). For our blackbuck videos (see “Collective behaviour of blackbuck herds” section for data description), even though we are sampling background examples (“no” class) from a majority of videos, we use a small proportion of frames from each video and owing to the heterogeneity of the background not all elements of the background are covered in training samples. Hence, when the network runs over the full video it encounters numerous regions in each frame that are new to the network.

### Data description

**Figure 2:**
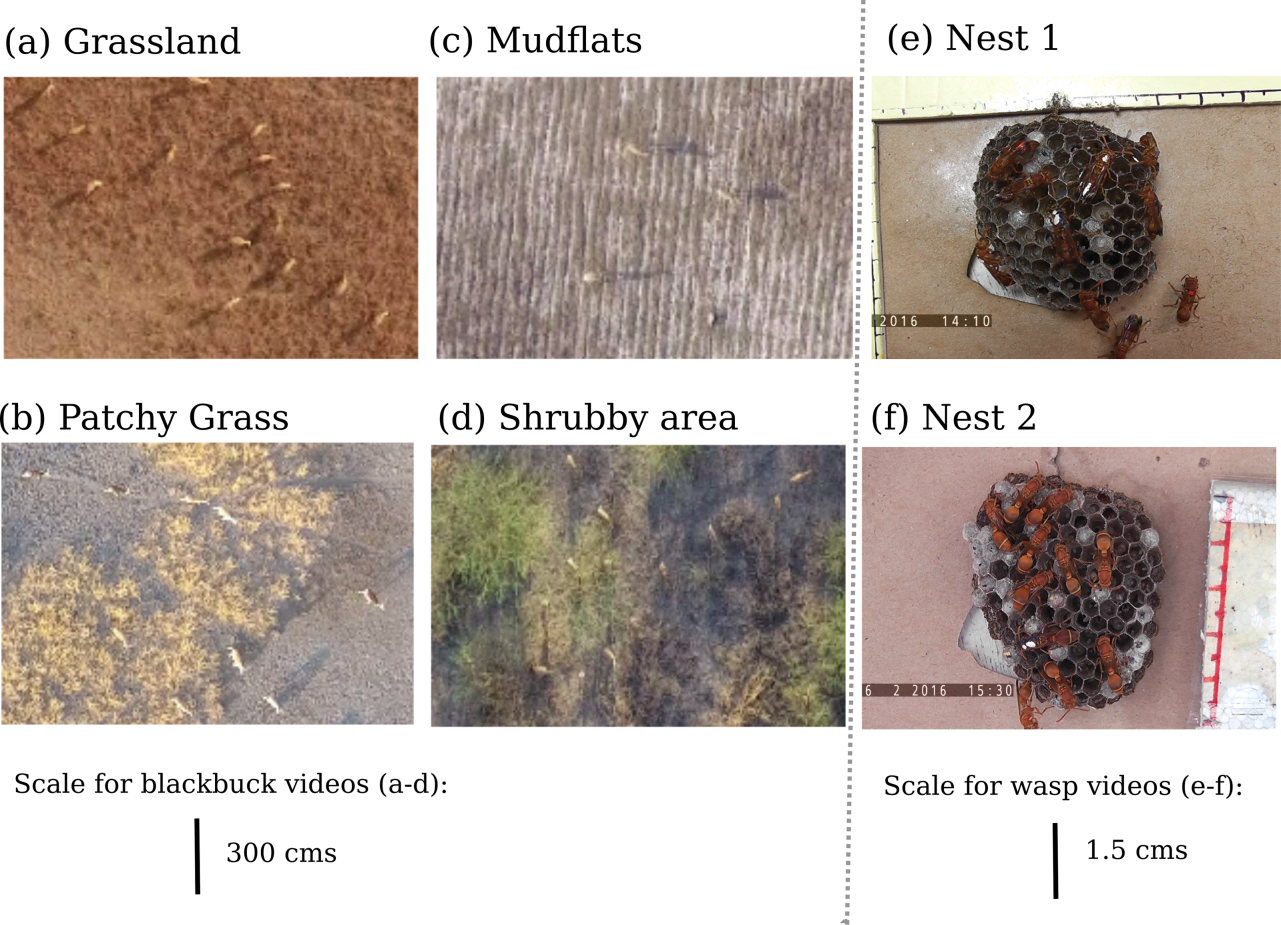
Variation in the appearance of animals and background in different videos. Variation in the appearance of animals and background in different videos: blackbuck herds in a (A) grassland, (B) habitat having patches of grass, (C) mudflat area of the park, (D) bush dominated habitat. Wasp nest with a majority of (E) older wasps, (F) newly enclosed wasps.

**Figure 3:**
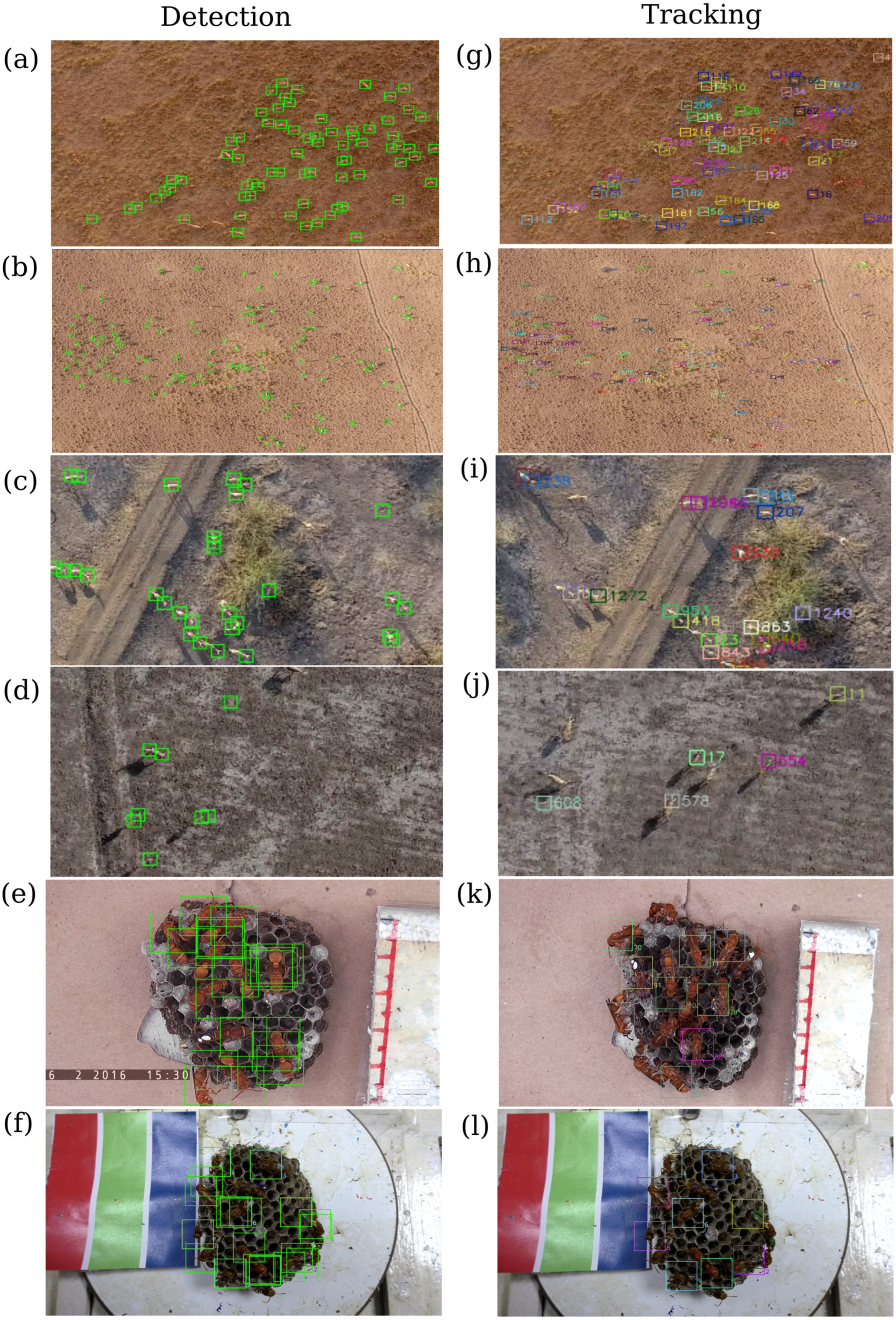
Detection and Tracking results in six example videos. (A) and (G) Moderate size blackbuck herd in a grassland; (B) and (H) A big herd (blackbuck—158 individuals) in the grassland; (C) and (I) blackbuck herd in a shrubby area; (D) and (J) blackbuck herd in the mudflats; (E) and (K) Nest with a majority of older wasps and (F) and (L) Nest with a majority of newly eclosed wasps. Each individual is assigned a unique number and colour after the tracking. All images are zoomed and scaled at different levels for visibility. The size of wasps is around 1 cm and blackbuck is around 1 meter.

To demonstrate the usage of the MOTHe application, we use videos of two species—blackbuck (*Antilope cervicapra*) and a tropical paper wasp (*Ropalidia marginata*). These two species present different types of complexity in terms of the environment (natural and semi-natural settings), background, animal speed, behaviour and overlaps between individuals (Fig. 2). The blackbuck videos were recorded in four different habitat types and the wasp videos on two different nests. The sample videos were all 30 s long. The maximum number of individuals present in these videos is 156 and 12 for blackbuck and wasps, respectively (Fig. 3). Below, we provide a description of these datasets and describe the steps to implement MOTHe (see Fig. 1 for an overview).

#### Collective behaviour of blackbuck herds

We recorded blackbuck (*Antilope cervicapra*) group behaviour in their heterogeneous natural habitat using unmanned aerial vehicles. Blackbuck herds exhibit frequent merge-split events (Mungall, 1978). These herds consist of adult males & females, sub-adults and juveniles (Isvaran, 2007; Isvaran, 2005). They are sexually dimorphic and the colour of adult males also changes with testosterone levels (Ranjitsinh, 1982). This colour variation makes it difficult to use colour segmentation-based techniques to detect them. The major source of complexity in analysing aerial videos of this system arises from their heterogeneous habitat, comprising semi-arid grasslands with patches of trees and shrubs. While many blackbuck do not move across many video frames, there is substantial movement of grasses and shrubs in the background. These conditions pose challenges for applying basic computer vision methods such as colour thresholding and image subtraction. For our aerial recordings, we chose different habitat patches such as grasslands, shrublands and mudflats in Blackbuck National Park, Velavadar. These recordings were made using a DJI quadcopter flown at a height of 40–45 m (Phantom Pro 4) equipped with a high-resolution camera (4 K resolution at 30 frames per second). The average size of an adult blackbuck is 120 cm from head to tail which corresponds to around 35 pixels in our videos. Throughout the recording sessions, each of which typically lasted for around 15 min, the animals continued their natural activity unperturbed by our aerial videography observations. The data collection was approved by the Office of the Principal Chief Conservator of Forests, Gujarat, India, Permit letter WLP/28/C/74-75/2016-17.

#### Nest space-use by wasps

We used videos of tropical paper wasps *Ropalidia marginata* recorded under semi-natural conditions (Sharma & Gadagkar, 2019). Here, individuals were maintained in their natural nests in laboratory conditions and were allowed to forage freely. Nests of *Ropalidia marginata* are sites for social interactions between mobile adults as well as between adults and immobile brood (Gadagkar & Joshi, 1983). These nests are made of paper, which offers a low contrast to the dark-bodied social insects on the nest surface. The nest is comprised of cells in which various stages of brood are housed and thus add to the heterogeneity of the background. Additionally, different nest colonies differ in the age composition of individuals, contributing to the variation in the appearance of wasps across videos. Therefore, this system too presents challenges to classical computer vision methods used to detect animals from the background. Recordings were done using a video camera (25 frames per second). The size of the wasp is 1 cm from the head to the abdomen which corresponds to around 150 pixels in our videos.

For blackbuck and wasp datasets, we acquired the necessary approval from the office of the Principal Chief Conservator of Forests & Head of The Forest Force (Government of Gujarat, India) and the Ethics Committee at the Indian Institute of Science, Bengaluru, respectively.

### Implementation on new datasets

MOTHe can be used to train a neural network and run the trained network for detection tasks on new videos. For any new set of videos, the user needs to run four modules of MOTHe described in the “Working principle & features section”: Generation of the training dataset, Network training, Detection and Track linking sections. We first recommend that the users set up MOTHe in their system and test it on the given sample videos to get acquainted with the MOTHe pipeline and functions. Detailed guidelines are available on the MOTHe-GUI repository. Once the user is familiar with the package, the next step is to generate the training data.

For any new application, “*How much training data do I need?*” is always a difficult but important question to answer. Neural networks generally work well with a huge number of training samples (Abraham, 2005; Larochelle et al., 2009; Liu et al., 2016). However, the exact amount of data required for the training purpose depends on many factors such as variation in the appearance of animals, presence of other animals in the videos, clutter in the background, variation in the background, *etc*. The principle behind this approach is that the network should be trained with sufficient examples of the objects that it might encounter during the detection task (Logothetis et al., 1994; Keshari et al., 2018; Montserrat et al., 2017; Tajbakhsh et al., 2016). Broadly, one should select frames from various videos so as to get a good representation of the animals and background heterogeneity. For example, if a user has 50 videos, it will be a good idea to sample frames from the videos that have different types of habitats. If there are sexually dimorphic species, then obtain a nearly equal number of samples for both males and females; and almost equal numbers of background samples in the “no” category. However, if the videos have background clutter and varying background conditions, then the number of samples in the “no” category may be much more than the “yes” category *i.e.,* the samples of animal images. Another parameter to be taken care of while generating training data is the size of bounding boxes (Rezatofighi et al., 2019; Rajchl et al., 2016). We suggest the user generates images that can encapsulate the biggest animal in the videos.

We show the effect of changing training dataset size on model performance in detection in Fig. S2. The CNN model is trained on training datasets of blackbuck videos of varying sizes, and then the validation accuracy is calculated. The validation accuracy for each model is plotted against the size of the corresponding training dataset. We find that the validation accuracy saturates as a function of training samples, reaching an accuracy of 90% by around 20% training samples. Based on this, we recommend using at least several thousand image examples for the animal category. This number may need to be increased if the animal of interest shows a lot of variation in morphology. For example, to train the MOTHe on our blackbuck videos, we used 9,800 cropped samples for blackbuck (including males and females) and 19,000 samples for the background because the highly heterogeneous background that included grass, soil, rocks, bushes, water, *etc.*

In the case of small datasets available for the training, we recommend a couple of ways to increase the training accuracy: One is data augmentation, which is a way to increase the amount of training data by slightly modifying the existing data or creating synthetic data. it increases the training sample size and also helps with overfitting issues by bringing in variability in the training data (Taylor & Nitschke, 2018; Huang et al., 2019; Moreno-Barea, Jerez & Franco, 2020; Perez & Wang, 2017). The most common ways to apply data augmentation are either by modifying the existing image in the training dataset or by creating artificial data using generative adversarial networks. Another way to deal with the issue of a small training sample size is by using transfer learning methods (Torrey & Shavlik, 2010; Shaha & Pawar, 2018). In this method, we can use previously trained networks as a starting point for training a new dataset. It works very well in cases where networks are trained for a similar or broader category (Pires de Lima & Marfurt, 2019; Zhao, 2017; Kleanthous et al., 2022). For example, a network trained to identify all ungulates can be used as a starting point to build a network for detecting certain antelope species.

The next step is to run the CNN on the training data. MOTHe uses a combination of grayscale thresholding and CNN for localisation and classification respectively (Deng, Todorovic & Latecki, 2017; Lan et al., 2019). To identify the regions that could potentially have the animal of interest, we apply grayscale thresholds at the pixel level. So, one important customisation required for new types of videos is to provide grayscale threshold values. The aim of providing colour thresholds is to identify all the possible regions that could have the animal of interest. Therefore, we aim to choose thresholds in such a way that the results are biased towards false positives *i.e.,* it is desirable to get key points in the background rather than missing the key points on the animals. A trial and error approach can be used to set threshold values. For more details, read the Supplementary Material Section 2.1 “*Choosing colour thresholds*”. It might take various sessions of training to get a good validation accuracy (preferably above 99%). To improve the validation accuracy, the user can increase training samples by including extensive representations of the animals and background class.

Once the MOTHe is trained with desirable validation accuracy, we can now test it on the videos. For this, the user needs to run the detection function of the MOTHe GUI. To improve the detections, one may be required to go back to the training data generation and training steps.

## Results

We now present results after running the trained CNN on four sample videos of blackbuck herds, representing different habitat types and group sizes (Figs. 2A–2D) and two sample videos of wasps, representing two different colonies (Figs. 2E–2F). In Fig. 3, the first column shows the results of running object detection on these video clips and the second column displays the results after implementing track linking on the detections. Column B in Fig. 3 shows the unique colour and number-coded boxes around the individuals after track-linking. MOTHe does not automatically draw the colour-coded tracks in the output videos but these can be drawn using a standalone program and output CSV file. Please see Fig. S4A for an example schematic of how the trajectories may look after drawing the tracks. Users may refer to tracked videos to see the example output. We observe that the package is able to detect and track a large fraction of individuals in all types of habitats (Table 1). However, as expected, there are some errors in animal detection using MOTHe.

Our analysis (see Table 1) shows that MOTHe provides reasonable true positives (of 80% and above) and low false positives rates (close to zero in most videos; see the Supplementary Material Section 4 for methods of computing these). We emphasise that even if some animals were not detected in particular frames, they were detected in the subsequent frames. Therefore, all the wasps and blackbuck present in our video clips were tracked by MOTHe (see Supplementary Videos). In Table 1, we show the time taken to run detection on these video clips (Table 1) on an ordinary laptop (4 GB RAM with an Intel Core i5 processor); we find that the number of frames processed in one second ranged from 0.5 to 2.5. This efficiency can be improved considerably by running MOTHe on workstations, GPUs or cloud services. The details of parameterisation, steps associated with data generation and CNN training for wasp videos and blackbuck videos, are described in section “*Running MOTHe app*” of the Supplementary Material (also see the GitHub repository). In Figs. S1 and S2, we also report the precision *versus* recall graph and how the accuracy of detection changes with the threshold we apply to identify an animal detection. Further, we show how CNN performs better than standard computer vision techniques, in the Supplementary Material Section 4.

We also quantify the performance of our tracking module. We have calculated the track length (measured in seconds) for two videos each from blackbuck and wasp datasets. Tracking length was computed for all the individuals in these clips for a duration of 30 s and time was noted until the track ID changed for the first time. We also include the track length for the second ID within these 30-second windows. The initial ID for every individual is noted along with the time the individual is tracked with consistent IDs. In case of ID reassignment due to a mistrack, the new ID is noted along with the time the new ID persists. A key assumption made to define tracking metrics is that one ID change (and hence mistrack) is allowed. An individual is considered to have lost track after a second mistrack/ID change.

**Table 1:** Results after running MOTHe detection on blackbuck videos in various habitats and wasp videos in two colonies. Each video clip is 30 s in duration and these results are averaged over 30 frames spaced at 1 s for each video. % true positives (TP) shows the percentage of individuals that were correctly detected in a frame and % false positives quantifies the background noise identified as an animal. The percentage of missed animals, *i.e.*, false negatives, can be computed as 100 - TP. We report the computing efficiency when using an ordinary laptop (4 GB RAM with an Intel Core i5 processor) in frames processed per second.

**Video**	**Group size**	**Habitat**	**% True positives (TP)**	**% False positives (FP)**	**Run time** (Frames processed per sec.)
Blackbuck-1	28	Patchy grass	89.3	14.2	1.99
Blackbuck-2	78	Grass	83.1	0	0.82
Blackbuck-3	156	Grass	97.4	0.64	0.51
Blackbuck-4	34	Shrubs	91.4	0	2.44
Wasp-1	15	Colony with majority older wasps	86.6	0	1.11
Wasp-2	16	Colony with newly eclosed wasps	93.75	0	1.06

We present the median and mode time lengths for first and second IDs for all individuals in Fig. S4B. These metrics suggest that all the individuals for blackbuck and wasp datasets were faithfully tracked for the 30-second duration with a mean track length of 27 s for blackbuck and 21 s for wasp videos. These durations are useful for the analysis of many group-level metrics. For example, to compute group properties such as polarisation and group cohesion, we often need IDs preserved only for consecutive frames of movement, *i.e* for 1/30th of a second. To compute how specific individuals influence others, let us say in the context of escape from a predator, we may need longer tracks lasting several seconds to minutes. Even in such cases, bursts of escape do not last long. Our tracking package together with manual corrections could facilitate such analysis. Therefore, we argue that both the detection and the tracking we have obtained with MOTHe are reasonable for various types of analyses of collective motion. For more specific analysis requiring the IDs of animals for longer time frames a manual intervention may be required to reassign the IDS and deal with ID switches due to occlusions or cross-trajectories.

## Discussion

In this article, we present the integrated and ready-to-use package MOTHe which allows users to generate datasets, train a simple neural network and use that to detect multiple objects of interest in a heterogeneous background. We demonstrate the application of MOTHe in different habitat types for two species. Demonstrated videos differ in terms of animal species, their movement type, animal-background contrast and background heterogeneity. MOTHe is a modular and semi-automated object detection package that can potentially be used for animal videos in their natural conditions. Furthermore, MOTHe can be used to track objects on a desktop computer or a basic laptop.

### Strengths and weaknesses of MOTHe

The use of machine learning for classification enables MOTHe to detect stationary objects. This bypasses the necessity of relying on the motion of animals for the detection of animals (Risse et al., 2017a). MOTHe has various built-in functions and is designed to be user-friendly; advanced users can customize the code to improve the efficiency further. Alternative methods for object detection, such as You Only Look Once (YOLO) (Redmon et al., 2016) or region-based convolutional neural network (RCNN) (Ren et al., 2015; Girshick, 2015) that perform both localisation and classification, are expected to reduce error rates compared to our approach and do not require colour thresholding. However, these types of neural networks require access to high-specification GPUs. Using these kinds of specialised object detectors for animal tracking requires sufficient user proficiency to configure. In contrast, we argue that MOTHe can be used by researchers with relatively minimal programming knowledge.

Like many animal detection and tracking algorithms (Pérez-Escudero et al., 2014; Risse et al., 2017a; Mönck et al., 2018; Sridhar, Roche & Gingins, 2018; Rodriguez et al., 2018; Yamanaka & Takeuchi, 2018), MOTHe is incapable of resolving tracks of individuals in close proximity (usually, when less than one body length). There are formal ways to quantify this; for example, by quantifying the probability density of swaps as a function of proximity. Furthermore, one can compute recall, precision and accuracy measures of MOTHe (see the Supplementary Material Section 4). To preserve computational efficiency, we did not incorporate issues arising from a shaking camera in the MOTHe application. However, our drone videos of blackbuck herds do exhibit a minor amount of shaking due to winds, yet the MOTHe was capable of detecting and tracking animals. MOTHe can be further strengthened in combination with image stabilizing algorithms, or better tracking algorithms, to solve issues arising from camera vibrations. In our examples, the maximum number of individuals presented to the detection algorithm was 156. Over a period of several frames, all animals in the video were detected, although each frame may have a detection error.

### Related packages

We now discuss some of the related packages aimed towards multi-object tracking in the context of visual animal tracking of unmarked individuals. In Table 2, we list the features of MOTHe with some recent tracking solutions. As per the review of a large number of open-source animal tracking packages by Panadeiro et al. (2021), only a few of the packages could track multiple unmarked animals. Some of these packages/methods, which are state-of-the-art for multi-object tracking are IdTracker (Pérez-Escudero et al., 2014; Romero-Ferrero et al., 2019), Tracktor (Sridhar, Roche & Gingins, 2018), ToxTrack (Rodriguez et al., 2018), ABCTracker (Rice et al., 2020), Fish CNN-Tracker (Xu & Cheng, 2017), TRex (Walter & Couzin, 2021) and FastTrack (Gallois & Candelier, 2021). However, documentation of each of these packages suggests that these tools were developed and demonstrated only for laboratory/controlled settings where there is sufficient contrast between animals and the background.

Some of the applications that use a deep learning or CNN-based approach for detection and/or tracking seems promising in achieving the goal of visually tracking animals in natural settings (Bewley et al., 2016; Dell et al., 2014; Kellenberger, Tuia & Morris, 2020; Koger et al., 2023; Torney et al., 2019). In Table 2, we present a qualitative comparison of the recently developed packages that show potential for visual tracking of multiple unmarked animals in natural settings. TRex (Walter & Couzin, 2021) focuses on improving the tracking accuracy and speed for multiple animals in real-time. It is impressive in tracking up to hundreds of individuals and individual identification of approximately 100 unmarked individuals with high accuracy, speed and 2–10 times less memory than other existing tools for visual tracking. However, authors have not demonstrated for videos recorded in natural field conditions. Another state-of-the-art in this direction is various implementations of SORT (Bewley et al., 2016). The SORT method combines a CNN-based approach for detection to improve the tracking efficiency of the Kalman filter and the Hungarian algorithms. It is demonstrated to perform remarkably well for rapid movements such as dancers’ trajectories. Although this package too has not been demonstrated in the natural settings for animal tracking, we speculate that may have the potential for the same. However, there is no readily available package and pipeline that could be used by novice users.

**Table 2:** A summary of the existing tools for automated visual tracking of animals based on qualitative features: Installation, interface, environment, detection method, tracking method, dataset generation, animals tested on and any additional features. We compare MOTHe with a variety of different tools such as TRex (Walter & Couzin, 2021), AIDE (Kellenberger, Tuia & Morris, 2020), SORT (Bewley et al., 2016) and Koger 2023 (Koger et al., 2023).

	**TRex**	**SORT**	**AIDE**	**Koger et al.**	**MOTHe**
Installation mode	Command-based	NA	Web-based	NA	Command-based
Integrated pipeline?	Yes	No	No	No	Yes
GUI	Yes	No	Annotation tool	No	Yes
Supported OS	Windows, Linux, Mac	NA	Web-based	NA	Windows, Linux, Mac
Image acquisition	Video input using TGrabs	Automated	Camera trap dataset	Model-assisted labeling	Point and Click
Detection method	Background Subtraction and Neural Networks	FrCNN	Deep learning	Detectron2 API within the PyTorch framework	Grayscale Thresholding, Deep Learning (using CNNs)
Tracking method	Kalman Filter and custom tree-based method for ID	Kalman Filter and Hungarian algorithm	Not supported	Modified version of the Hungarian algorithm	Kalman and Hungarian algorithms
Animals tested	Fish and Insects	Not tested on animal videos	NA	Monkeys and African ungulates	Antelope and Wasp
Demonstration for natural conditions	No	No	NA	Yes	Yes
Max #animals	100	NA	NA	1024	156
Manual Id correction required?	No	Maybe	NA	Maybe	Yes
Extra features	Posture analysis, 2D visual fields and real-time tracking			Body postures (poses) and environmental features reconstruction	

The focus is now shifting towards integrated solutions for detecting and/or tracking multiple animals in the wild. First in line is a recently developed tool–AIDE (Kellenberger, Tuia & Morris, 2020). AIDE is primarily an open-source web framework designed for image annotation for ecological surveys. It provides an easy-to-use and customisable labelling interface that supports multiple users, while also integrating machine learning models to train on annotated data. However, unlike MOTHe, it does not provide a graphical user interface for detecting and tracking multiple animals in the wild. The recent work by Koger et al. (2023) demonstrates a solution for recording and visually tracking animals in the wild along with additional features such as posture estimation and habitat reconstruction. It also discusses the challenges in acquiring and processing such data to study animal behaviour and potential ways to minimize the complications at the data processing level. It uses and builds on the existing deep learning methods for animal detection, namely, Detectron2 API within the PyTorch framework. However, the authors of the article also concede that coding skills and specific computing environments are necessary to implement and customize this method. Specifically, some knowledge of Python programming is required to attune the parameters and modify code for a new dataset; in contrast, for MOTHe we provide a GUI interface for all the steps relevant for visual tracking on a new video dataset.

Ferreira et al. (2020) proposed a CNN-based deep learning tracking tool for individual recognition of birds in semi-natural settings (birds kept in cages outdoors). However, unlike our context where animals are unmarked, the individual birds were fitted with PIT-tags and the feeders were fitted with RFID antennas. Tags and information from RFID were used during the labelling and training stage of the CNN model. Furthermore, they largely focused on videos consisting of one bird, with some cases of a small flock size consisting of up to the three birds only. Hence, it is unlikely that this application will be suitable for large herd datasets that our package focuses on. In summary, in comparison with other packages for multi-object tracking MOTHe’s strength lies in an integrated and ready-to-use GUI-based pipeline for animal tracking in natural settings, where the background is heterogeneous and may change both within and across the videos. In addition, MOTHe also automates various steps related to object tracking such as data generation, test and training modules with click-and-execute functionality making it relatively easily accessible to field biologists and ecologists.

### On open source packages

Our work contributes to a growing body of open-source packages that implement deep learning for animal detection and tracking in the wild. However, there are many challenges as well as opportunities associated with open-source packages (Nolden et al., 2013; Ven, Verelst & Mannaert, 2008; Miller, Voas & Costello, 2010; Appelbe, 2003). Some of the pros of open-source packages are that they are free to use and can be customized to specific applications. The vibrant user community often actively contributes, leading to rapid updates and novel features. On the other hand, they are not always user-friendly and require manual installations and upgrades with no customer support services. The subscription-based software, on the other hand, overcomes these limitations by being user-friendly, tailoring for specific contexts and offering customer support. However, since the code is not publicly available, it may not be feasible to customise them to new contexts at all. Furthermore, subscription fees may make the tool inaccessible to a large part of the scientific community, especially those from lower and middle-income countries.

Within the open-source scientific community, there is often a focus only on developing newer methods rather than making an integrated solution available to novice users who do not have a programming background. In this context, we argue that MOTHe contributes to open-source animal detection and tracking packages by balancing technical methods and specificity of the application while focusing on user-friendliness—an aspect often overlooked.

## Conclusion

Over the past few years, there have been several encouraging developments in machine-learning-based tools to analyse drone or UAV-based videos of animals taken under natural conditions (Corcoran et al., 2021; Kellenberger, Tuia & Morris, 2020; Kellenberger, Marcos & Tuia, 2018; Rey et al., 2017; Torney et al., 2018). These methods show promise in reducing biases prevalent in ground-based surveys and improve the accuracy of detection of animals. They are useful for applied questions such as biodiversity surveys, as well as for answering fundamental ecological questions, such as how animals move, aggregate, and find mates in natural habitats (Koger et al., 2023; Rathore, Isvaran & Guttal, 2023). In this context, we hope that MOTHe offers a relatively user-friendly tool for researchers to track stationary as well as moving animals in their natural habitats. Users interact with a graphical interface at each step of the detection and tracking process. MOTHe is available as an open-source repository, complete with a detailed user guide and demonstrations on GitHub. We believe that this end-to-end package will encourage more researchers to use video observations to study animal group behaviour in natural habitats and will be of use to a larger research community.

We hope our work encourages further work on developing better algorithms for the detection and tracking of animals for videos taken in natural field conditions. Future studies could evaluate the performance of different methods, including MOTHe, under different types of natural conditions. More broadly, we call for the development of multi-object tracking tools that are easy to use by non-experts and which can be deployed using relatively limited computational resources; these aspects are sometimes overlooked while developing state-of-the-art digital tools for ecological contexts (Sethi, Evers & Balakrishnan, 2023). We also argue that a diversity of open-source methods and tools will facilitate the use of UAV-based imaging for ecological studies in various contexts, such as herding, lekking, and conservation ecology.

##  Supplemental Information

10.7717/peerj.15573/supp-1 Supplemental Information 1 Supplementray figures and methods Supplementary document containing figures and methods

## Additional Information and Declarations

### Field Study Permissions

The following information was supplied relating to field study approvals (i.e., approving body and any reference numbers): The Office of the Principal Chief Conservator of Forests, Gujarat, India, approved the study (Permit letter WLP/28/C/74-75/2016-17). This letter approved our application to use drones to record aerial videos of blackbuck leks. We conducted behavioral observations to ascertain that drones had minimal or no impact on blackbuck and they didn’t perceive drones as a threat.

### Data Availability

The following information was supplied regarding data availability: The sample tracked and untracked videos are available at Figshare: Rathore, Akanksha; Sharma, Ananth; Sharma, Nitika; J. Torney, Colin; Guttal, Vishwesha (2020): Multi-Object Tracking in Heterogeneous environments (MOTHe) for animal video recordings. figshare. Media. https://doi.org/10.6084/m9.figshare.11980356.v3
The sample clips that can be used to replicate detection and tracking using MOTHe in the MOTHe package is available at GitHub and Zenodo: https://github.com/tee-lab/MOTHe-GUI
Akanksha Rathore, & Vishwesha Guttal. (2021). tee-lab/MOTHe-GUI: MOTHe-GUI (MOTHe). Zenodo. https://doi.org/10.5281/zenodo.5582965.
