# Supplementary material for: Multi-Object Tracking in Heterogeneous environments (MOTHe) for animal video recordings
Source: PeerJ. 2023 Jun 26;11:e15573. doi: 10.7717/peerj.15573 (PMC10309051; doi:10.7717/peerj.15573)
Supplement: Supplemental Information 1 — Supplementary document containing figures and methods [file peerj-11-15573-s001.pdf]

Supplementary Material: Multi-Object Tracking in Heterogeneous environments (MOTHe) for animal video recordings

Akanksha Rathore[1,†], Ananth Sharma[1], Shaan Shah[4], Nitika Sharma[1,2], Colin J. Torney[3], and Vishwesha Guttal[1,†]

[1]Centre for Ecological Sciences, Indian Institute of Science, Bengaluru, India.
[2]Department of Ecology and Evolutionary Biology, University of California, Los Angeles, USA
[3]School of Mathematics and Statistics, The University of Glasgow, Glasgow, UK
[4]Department of Electrical Engineering, Indian Institute of Technology Bombay, Mumbai, India
[†]Correspondence may be addressed to: rathore.aakanksha58@gmail.com or guttal@iisc.ac.in

April 21, 2023

Contents

1 Pipeline Description **1**
1.1 Region proposal method . . . 2
1.2 CNN architecture . . . 2

2 Parameterisation **3**
2.1 Choosing colour thresholds . . . 3

3 Data generation and CNN Training **4**

4 Model Evaluation **4**
4.1 Tracking performance . . . 7

5 Permission to use Wasp videos **10**

1 Pipeline Description

MOTHe can automate all the tasks associated with object classification and is divided into five modules dedicated to the following tasks.

1. **System configuration**: The system configuration is used to set up MOTHe on the user's system. Basic details such as the path to the local repository, the path to the video to be processed, the size of the individual to be cropped and the size of the bounding box to be drawn during the detection phase.

2. **Dataset generation**: The dataset generation is a crucial step towards object detection and tracking. The manual effort required to generate the required amount of training data is huge. The data generation class and executable semi-automate the process by allowing the user to crop the region of interest with simple clicks over a GUI and automatically save the images in appropriate folders. For our models, we used roughly 9k animal images and 18k background images. These images were selected from a subset of frames from all the videos (to be able to cover various background contexts and animal representations). We selected roughly 15 sparsely spaced frames from 45 videos and cropped animals and background regions to generate the training data.

3. **Training the convolutional neural network**: After generating a sufficient number of training examples, the data is used to train the neural network. The neural network produces a classifier as the output. The accuracy of the classifier is dependent on how well the network is trained, which in turn depends on the quality and quantity of training data. The various tuning parameters of the network are fixed to render the process easy for the users. This network works well for binary classification - the object of interest (animals) and background. Multi-class classification is not supported in this pipeline. We divided the data generated using the previous step into training and validation data in a 4:1 ratio.

4. **Object detection**: This is the most crucial module in the repository. It performs two key tasks - it first identifies the regions in the image which can potentially have animals, this is called localisation; then it performs classification on the cropped regions. This classification is done using a small CNN (6 convolutional layers). Output is in the form of *.csv* files which contains the locations of the identified animals in each frame. The trained network was then applied to all the frames of the videos to get the detections.

5. **Object tracking**: Object tracking is the final goal of the MOTHe. This module assigns unique IDs to the detected individuals and generates their trajectories. We have separated detection and tracking modules so that they can also be used by someone interested only in the count data (eg. surveys). This modularisation also provides flexibility in using more sophisticated tracking algorithms for experienced programmers. We use an existing code for the tracking task - https://github.com/ctorney/uavTracker. This algorithm uses Kalman filters and the Hungarian algorithm. This script can be run once the detections are generated in the previous step. Output is a *.csv* file which contains individual IDs and locations for each frame. Video output with the unique IDs of each individual is also generated.

1.1 Region proposal method

To perform the detection task, we first need to identify the areas in an image where the object can be found, this is called *localisation* or *region proposal*. Then we classify these regions into different categories (e.g. whether an animal or background?), this step is called *classification*. The localisation step is performed using an efficient thresholding approach that restricts the number of individual classifications that need to be performed on the image. As discussed earlier, the colour thresholding approach doesn't perform well in complex videos and in most cases, there is a trade-off between false positives and missed identifications. To utilise colour thresholding for region proposal, we err on the side of false positives so that we get all the potential regions where an animal can be found. These identified keypoints are used to run the classification step.

1.2 CNN architecture

We selected the network architecture using a trial-and-error approach on several networks having 6-8 convolutional layers and varying parameters for these layers. We also explored different parameters for activation functions and dropout layers. Out of these networks, the best-performing architecture on the validation dataset was chosen. Here, we explain the architecture of MOTHe.

Our CNN consists of three convolutional and two dense layers. The number of nodes for convolutional layers is 64, 96 to 128 and for dense layers is 96 and 128. On the top of each convolutional layer, we use an activation function and a pooling layer. Dropout layers are added to avoid overtraining.

Below we list the layers and parameters of MOTHe-

(i) **Convolutional layer** - (nodes = 64, kernel size = (3, 3), activation='linear', input shape=(40,40,3), padding='same')

(ii) **Activation function** - LeakyReLU(alpha=0.1)

(iii) **Pooling layer** - MaxPooling2D ((2, 2),padding='same')

(iv) **Convolutional layer** - (nodes = 96, kernel size = (3, 3), activation='linear', padding='same')

(v) **Activation function** - LeakyReLU(alpha=0.1)

(vi) **Pooling layer** - MaxPooling2D((2, 2),padding='same')

(vii) **Dropout function** - (25 %)

(viii) **Convolutional layer** - (nodes = 128, kernel size = (3, 3), activation='linear', padding='same')

(ix) **Activation function** - LeakyReLU(alpha=0.1)

(x) **Pooling layer** - MaxPooling2D((2, 2),padding='same')

(xi) **Dense layer** - (nodes = 96)

(xii) **Activation function** - LeakyReLU(alpha=0.1)

(xiii) **Dropout function** - (30 %)

(xiv) **Dense layer** - (nodes = 128)

(xv) **Activation function** - LeakyReLU(alpha=0.1)

(xvi) **Dropout function** - (30 %)

2 Parameterisation

For ease of use, we have kept the parameterisation process minimal. Therefore, the various tuning parameters of the network architecture are fixed. However, advanced users can change the parameters in the code to customise it for more sophisticated tasks. The only step which requires some amount of parameter scanning by users is choosing the *colour thresholds* for animals. As mentioned earlier, to improve processing speed, we use a colour thresholding approach as the localisation step and select the thresholds in a way that we get all the potential regions where animals might be present (i.e. allowing the false positive cases). For this technique, users need to input the values of the minimum and maximum threshold of the pixels that may potentially correspond to the animal. To choose the values for any generic dataset, we provide detailed instructions under the section *Choosing colour threshold* (section 2.1).
The parameters for our two datasets are:
Blackbuck videos: min threshold = 0, max threshold = 150
Wasp videos: min threshold = 150, max threshold = 250

2.1 Choosing colour thresholds

The object detection steps require a user to enter threshold values in the config files. Object detection in MOTHe works in two steps, it first uses a colour filter to identify the regions in the image on which to run the classification. We use a colour threshold to select these regions. You can see the values of thresholds for blackbuck and wasp videos in the *config.yml* file. If you are running MOTHe on a new dataset, there are two ways to select appropriate threshold values:

1. You may open some frames from different videos in an interactive viewer (for example MATLAB image display), you can then click on the pixels on the animal and check the RGB values (take the average of all 3 channels). Looking at these values in multiple instances will give you an idea to choose a starting minimum and maximum threshold. Run the detection with these thresholds and you can improve the detection by hit and trial method to tweak the threshold.

2. You can compare your videos to wasp and blackbuck videos and start with threshold values to which your data is more similar. For example, if your animal looks more similar to blackbuck in colour and lighting conditions, you may start with default thresholds and improve the detection by changing the lower and upper threshold by a small amount at a time.

3 Data generation and CNN Training

To generate data for training the CNN, we run the function ”generate data” in the MOTHe-GUI app which then displays frames from the videos; for each of these frames, we select animals and background examples that are used in the training step. The resulting output is automatically stored in separate folders for animals and backgrounds.

To generate training samples for blackbuck videos, we used 2000 frames from 45 videos; these videos were recorded in different habitats. The number of individuals in these videos ranges from 30-300 individuals. We fixed the values of the grey-scale threshold for blackbuck to be [0,150]. We then run the CNN training function.

Likewise, to generate data and train the network with features of wasps we used equally spaced 1000 frames from 6 different videos. We fixed the values of the grey-scale threshold for wasps to be [150,250].

4 Model Evaluation

The working of MOTHe was tested out by using it to track Blackbucks in aerial footage shot in the wild. The performance of the CNN (which is used to detect the presence of an animal in a cropped section of the frame) was measured by calculating parameters such as precision, recall and accuracy.

1. Precision: It measures the tendency of the model to predict a false positive. The higher the precision value, the lower the model's tendency to predict a false positive.

$$Precision = \frac{True\ Positives}{True\ Positives + False\ Positives}$$

2. Recall: It measures the tendency of the model to predict a false negative. The higher the recall value, the lower the model's tendency to predict a false negative.

$$Recall = \frac{True\ Positives}{True\ Positives + False\ Negatives}$$

3. Accuracy: It measures the overall performance of the model. A higher accuracy signifies better performance of the model on the dataset.

$$Accuracy = \frac{True\ Positives + True\ Negatives}{True\ Positives + False\ Positives + True\ Negatives + False\ Negatives}$$

The performance of the CNN can be tuned by changing the threshold probability using which a prediction is made, i.e. if the probability of a “positive” (animal is present in the cropped version of the frame which is predicted by the model) is greater than the threshold probability, then the prediction of the model is taken to be a “positive” else it is taken to be a “negative”.

So it can be seen that on increasing threshold probability, the lower will be the number of false positives, which will lead to higher precision and the higher will be the number of false negatives, which will lead to a lower recall. On decreasing the threshold probability, the precision will fall due to higher false positives and the recall will rise due to lower false negatives. This is called the “precision/recall tradeoff”. The precision/recall tradeoff has been plotted in Fig 1-(a) and the accuracy of the model has been plotted against threshold probability in Fig 1-(b). The threshold probability is used to tune the performance of the model. If the predicted probability (by the CNN) of an animal being present in a frame is greater than the threshold probability then the prediction is taken to be a “positive” (animal is present in frame), else it is taken to be as a “negative” (animal is not present in frame). The accuracy is calculated as a ratio of the correct predictions and the total test cases.

To further access the performance of MOTHe, we compare the output of MOTHe with two standard computer vision techniques used for image segmentation, colour thresholding and image subtraction, on 10 videos each from wasp and blackbuck datasets. We compute failed and false detection errors. To quantify these error rates, we prepared *ground-truth* data by visually counting the number of individuals present in each frame and compared it with the number of detections obtained by running MOTHe; this was repeated for 30 frames spaced at 1 second and for each of the videos. This quantification gives us ground-truth values of animals and detections. We then compared this with the detections of animals using the MOTHe on the same set of frames to obtain (i) the percentage of true detections and (ii) the percentage of false detections (i.e. arising from the wrong classification of

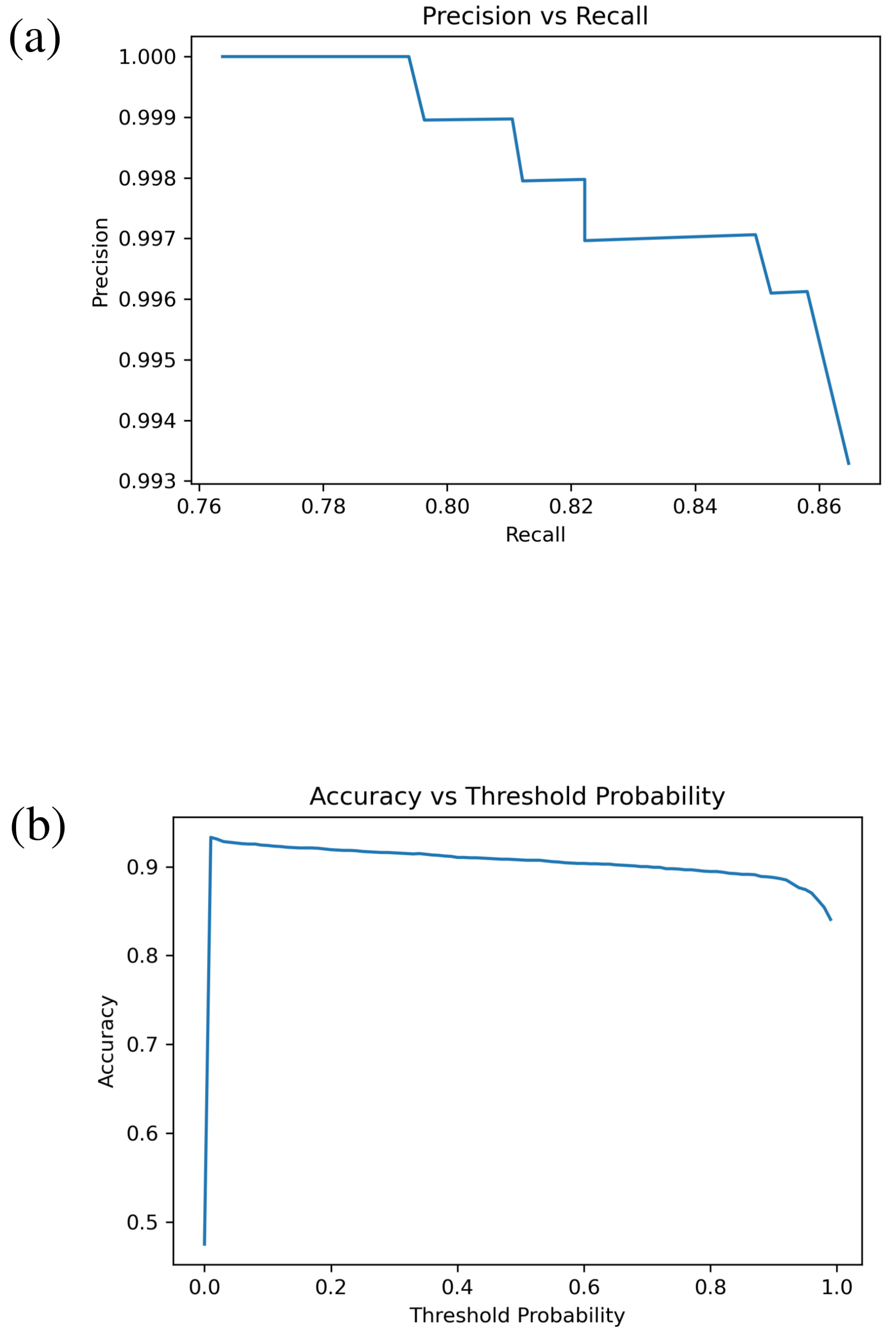

Figure 1: (a) The precision and recall values for the model have been plotted by changing the threshold probability. The precision value measures the tendency of the model to predict false positives and the recall measures the tendency of the model to predict false negatives. (b) The accuracy in the detection of an animal (using the CNN) in a cropped section of the frame has been plotted against the threshold probability.

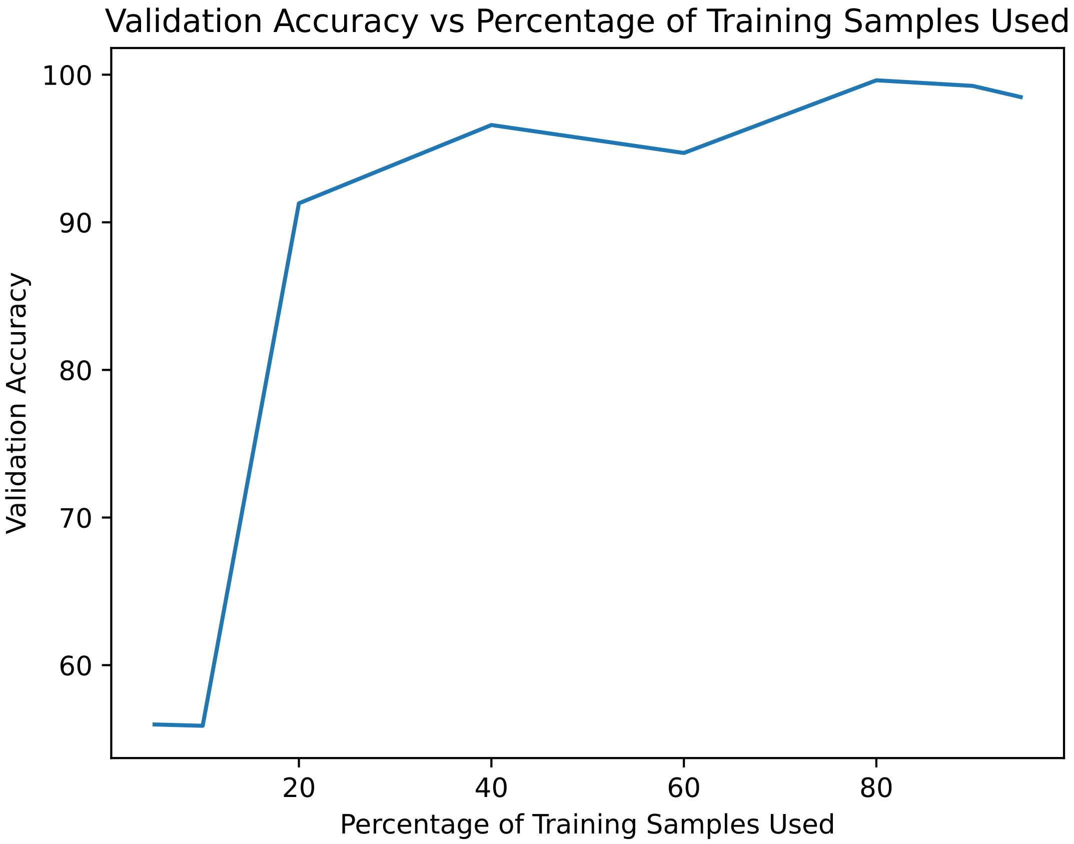

Figure 2: The validation accuracy has been plotted against the percentage of samples used for training the model. It can be seen that increasing the training dataset size up to a certain point leads to rapid gains in accuracy, after which the performance improvement is incremental.

background objects as animals).

We compare the performance of all three techniques by quantifying false and failed detection errors (Fig 3-(b)). It is interesting to see that MOTHe (CNN-based technique) is the only technique which provided good detections for blackbuck videos which are quite complex in terms of background heterogeneity and colour contrast. Blackbuck videos have many other challenges such as the similar colour of animals and background, movement of background objects such as grass, shrubs and other animals and stillness of many blackbuck individuals over a long duration. The animal and background similarity makes it difficult to apply the colour thresholding approach and movement in background and animal stillness, making it difficult to get detections using image subtraction.

4.1 Tracking performance

Since MOTHe is aimed towards providing a complete user-friendly setup for the steps related to visual animal tracking in videos, we also quantify the performance of our tracking module. We have calculated the track length (measured in seconds) for two videos each from blackbuck and wasp datasets. Tracking length was computed for all the individuals in these clips for a duration of 30 seconds and time was noted until the track ID changed for the first time. We also include the track length for the second ID within these 30-second windows. The initial ID for every individual is noted along with the time the individual is tracked with consistent IDs. In case of ID reassignment due to a mistrack, the new ID is noted along with the time the new ID persists.

A key assumption made to define tracking metrics is that one ID change (and hence mistrack) is allowed. An individual is considered to have lost track after a second mistrack/ ID change. We present the median and mode time lengths for first and second IDs for all individuals in Figure 4-(b). These metrics suggest that all the individuals for blackbuck and wasp datasets were faithfully tracked for the 30-second duration with a mean track length of 27 seconds for blackbuck and 21 seconds for wasp videos.

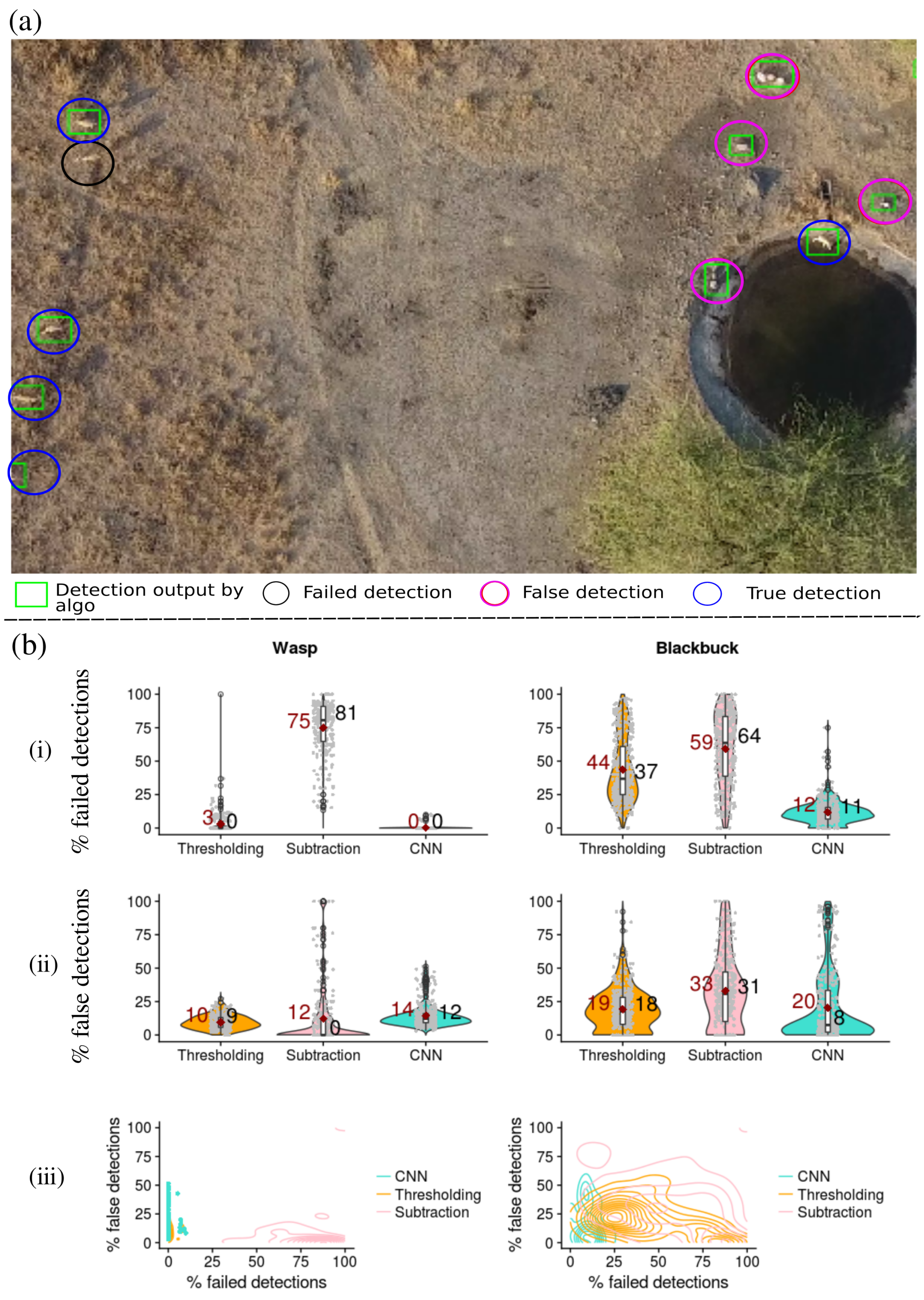

Figure 3: (a) A schematic of the output from different methods and examples of failed, false and true detections. (b) Comparison of the performance of all three object detection techniques on both species' videos.(i), (ii) & (iii) represent the % failed (missed) detections, false (noise) detections and failed & false error distributions for wasps and blackbuck respectively.

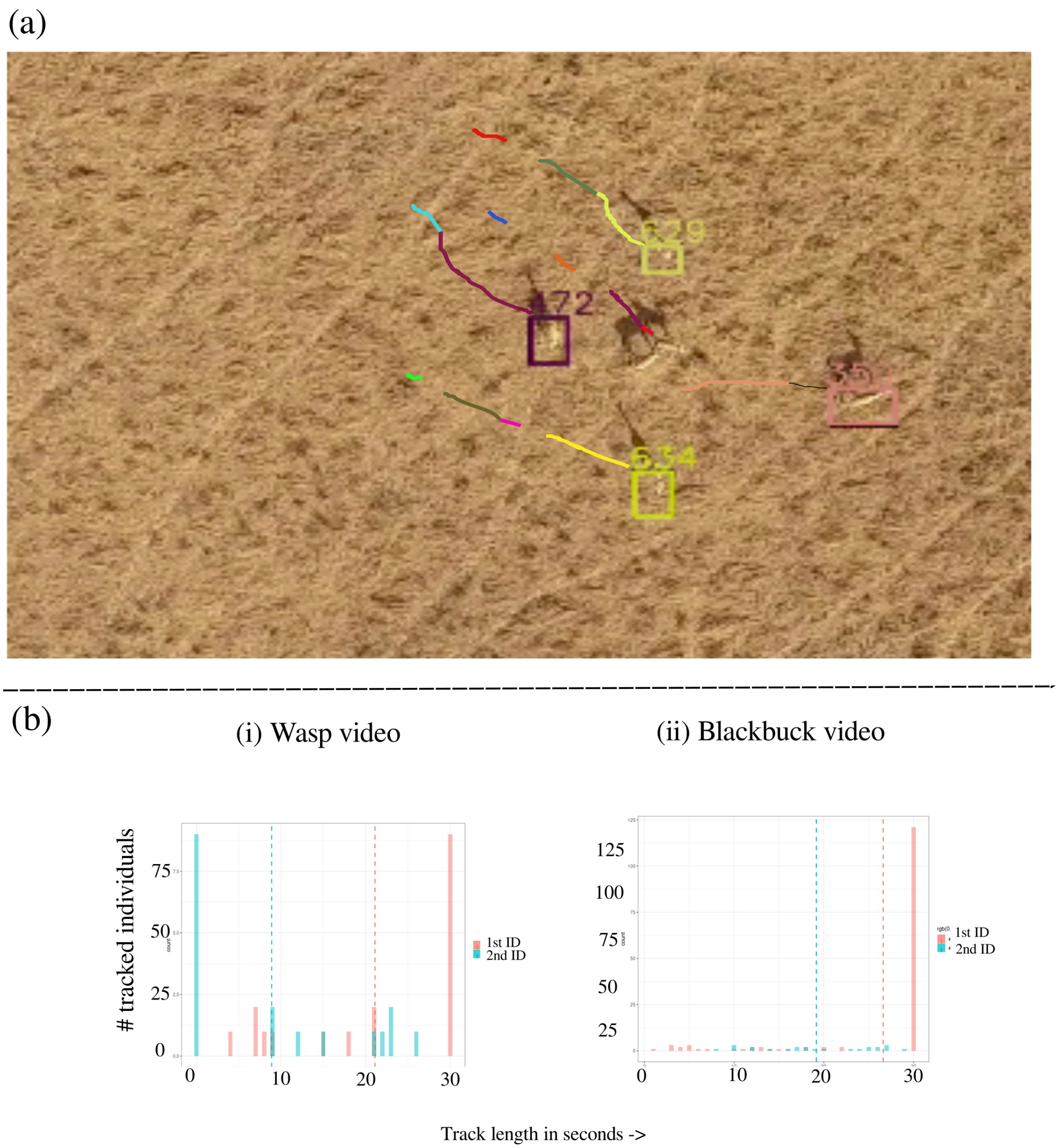

Figure 4: (a) An example of the tracks that could be generated using the coordinate values from the output file. MOTHe generates tracking results in the form of individual IDs and x-y coordinates. Each ID is uniquely represented by a number and colour value. Example trails in this figure represent the duration for which each individual was associated with a unique ID (marked by different colours in the figure). (b) Track length calculated for one sample clip each of wasp and blackbuck. We calculated the time for which the 1st and 2nd assigned IDs lasted for all the individuals in these videos.
